# Influence of premorbid BMI on clinical characteristics at presentation of adolescent girls with eating disorders

**DOI:** 10.1186/s12888-016-0788-7

**Published:** 2016-03-31

**Authors:** Ingemar Swenne

**Affiliations:** Department of Women’s and Children’s Health, Uppsala University Uppsala, S-75185 Uppsala, Sweden

**Keywords:** Eating disorder, Adolescent, Premorbid weight, Overweight, Obesity

## Abstract

**Background:**

Considering the prevalence of obesity in society it can be expected that some adolescents with an eating disorder (ED) start weight loss from an overweight and present at a near-normal weight. Presently, the influence of premorbid BMI on clinical characteristics of adolescent girls presenting with an ED has ben studied.

**Method:**

Premorbid growth charts were available for 275 postmenarcheal adolescent girls presenting with an ED (anorexia nervosa = 27, (subthreshold) bulimia nervosa = 9, restrictive EDNOS = 239). Initial assessment included measurement of weight and length, physical examination, blood sampling and administration of the Eating Disorder Examination-Questionnaire youth version (EDE-Q).

**Results:**

Despite greater weight loss girls with a high premorbid body mass index (BMI) had a higher BMI at presentation compared to those with a lower premorbid BMI. Although not underweight some presented with clinical and laboratory signs of starvation. These signs were related to not only low BMI but also to rapid and large weight loss. Their EDE-Q scores did not differ from those of girls who presented with an underweight.

**Conclusion:**

Girls with a restrictive ED and premorbid overweight may present with a near-normal BMI. They can nevertheless be medically compromised and have eating disturbed cognitions at the level of underweight girls. They should not be regarded as having a less severe ED but merit full assessment and a start of treatment.

## Background

With overweight and obesity prevalent in society it must be expected that some adolescents falling ill with an eating disorder (ED) start weight loss from an overweight. With overweight there may even be an increased risk of developing an ED considering the relationship between obesity, body dissatisfaction and restrictive eating [1–4]. Body dissatisfaction and the wish to be thinner may be strong and attempts to diet, initially appraisable, may take their own course and develop into an ED.

It is an old observation that patients with anorexia nervosa (AN) often have a weight above average prior to the onset of weight loss [5]. This has also been observed in adolescents with restrictive ED not otherwise specified (EDNOS) for whom growth charts from the school health services could be used to document premorbid weight and height [6]. Adolescents with restrictive ED who start their weight loss from an overweight may have a long duration of ED symptoms but nevertheless present with higher BMI than those with a premorbid weight in the average range [7–10]. Despite considerable weight loss they do not reach the weight criterion for AN but fall into the EDNOS category. ED cognitions may nevertheless be in parity with those of underweight adolescents with ED [9–12]. Indeed, the difference between premorbid and current weight, often termed weight suppression [13], is an independent predictor of ED psychopathology [14]. These patients may present a considerable clinical challenge due to an intense fear of a weight gain which would return them to their previous overweight. If they maintain their weight suppression this may adversely influence outcome of the ED [14, 15]. Moreover, despite not being underweight they may have signs of medical instability and require medical intervention at the start of treatment [16, 17].

To further elucidate the relationship between premorbid BMI and clinical features at presentation of an ED we have analysed a sample of adolescents with ED and with objective measures of premorbid BMI available. In doing so we have included only postmenarcheal girls since those who have not completed puberty and reached menarche before onset of the ED have a different growth pattern [18]. It was hypothesized that adolescents who start weight loss at a higher BMI would not be as underweight at presentation as those with premorbid BMI closer to population average but that they would differ little in eating disturbed cognitions. It was also hypothesized that adolescents with rapid weight loss would present with clinical and laboratory signs of starvation, despite not being severely underweight.

## Methods

### Participants and procedure

The Child and Adolescent Eating Disorders Unit of Uppsala University Hospital is a specialised service offering treatment to all patients with an ED and <18 years of age in the county (population 335 882 of which 69 314 < 18 years on Dec 31 2010). Patients are initially assessed by a paediatrician according to a structured protocol including the history of the ED, demographic and medical background data, a physical examination, and blood sampling. Referral is usually by the school health services or following a direct contact with the hospital by the family. Patients are seen without delay, usually within 2 weeks following receipt of referral. With few exceptions the patients have not been treated for their ED prior to the initial assessment. An eating disorder diagnosis is established and parents are informed that start of treatment aims at stopping weight loss and normalising eating by parents taking charge of meal routines and advice is given on how to start this [19]. A first follow-up is 1 week later at which diagnostic interviews are completed, self-report instruments administered and a diagnosis according to the Diagnostic and Statistical Manual of Mental Disorders, 4th edition (DSM-IV) finalized. For the present investigation patients were retrospectively reclassified according to the criteria of DSM-5.

During the period August 2010–August 2015 355 adolescents were assessed for an ED. The present study is an analysis of data from 288 postmenarcheal girls who had not previously been treated for their ED. All participants and their guardians consented to participating in the study. The protocol was approved by the Ethics committee of the Faculty of Medicine of Uppsala University.

### Study measures and statistics

Growth charts were procured from the school health services. A recorded premorbid maximal weight could be obtained for 275/288 (95 %) of the postmenarcheal girls and was a prerequisite for inclusion in the analyses. It had been reached less than a year before presentation for 185 (64 %). A prepubertal weight at approximately age seven was available for 231 (80 %). A recorded weight 5–63 days (24 ± 15 days; mean ± SD) prior to assessment was available in 246 (85 %). A family doctor or a nurse in the school health services had usually recorded this weight. Total weight loss was calculated as the difference between maximal recorded weight on the school growth charts and weight at assessment. Final rate of weight loss was calculated for the weight loss from the last recorded weight prior to assessment. Body mass index (BMI) was calculated as weight/height^2^ (kg/m^2^). BMI was recalculated into BMI standard deviation scores (BMI SDS), which constitutes a measure of leanness corrected for age and height [20]. Timing of menarche was by patients/parents recall at assessment and often corroborated by notes on the growth charts. Gynaecological age was calculated as the difference between age at assessment and age at menarche.

The self-report instruments Eating Disorders Examination-Questionnaire youth version (EDE-Q) [21], Montgomery-Åsberg Depression Rating Scale-Self report (MADRS-S) [22, 23] were administered to assess eating disturbed ideation and depressive symptoms, respectively.

Laboratory analyses of non-fasting blood samples were performed at the Department of Clinical Chemistry at the Uppsala University Hospital as part of the clinical work-up of the patients. Blood samples had been obtained for 268 (97 %) of the participants. The laboratory is certified by a Swedish government authority (Swedac). The correlation and stability of the assays were continuously reviewed. The total assay variation in the individual hormone assays was less than 5 %.

Patients were grouped by their premorbid BMI SDS into one SDS wide classes. Values are given as means ± SD. Differences in outcome variables between BMI SDS classes were compared using a one-sided ANOVA for continuous data and Chi-square test for categorical data. The relationship of BMI SDS and (rate of) weight loss with clinical and laboratory measures was analysed by a linear stepwise regression or by logistic regression. All analyses were performed with SPSS 20.0.0.

## Results

**Fig. 1:**
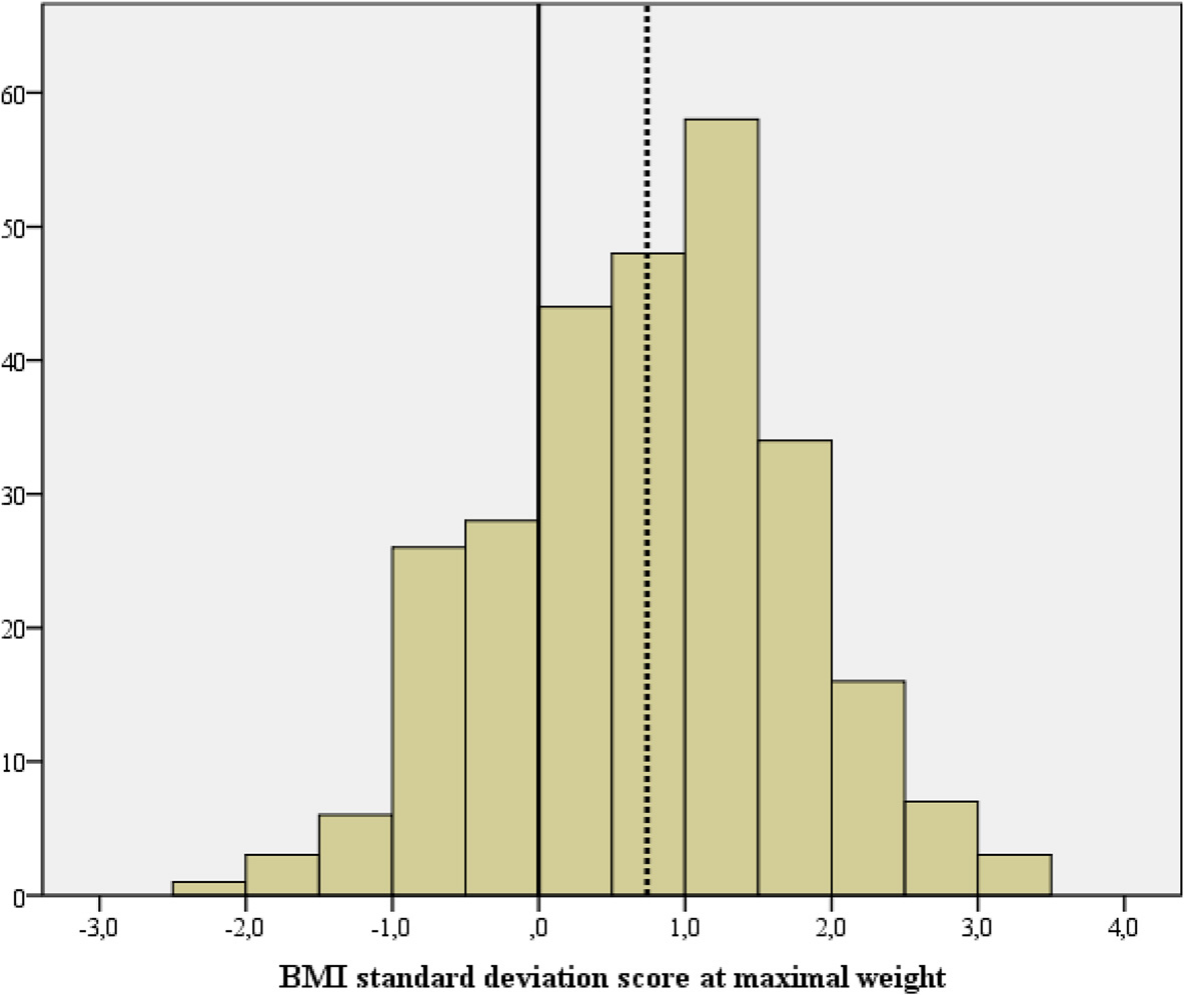
Body mass index (BMI) standard deviation score at the maximal premorbid weight of 275 postmenarcheal adolescent girls presenting with an eating disorder. BMI SDS = 0 (solid line represents the population average. The average of the present sample (BMI SDS 0,73 ± 1,00; mean ± SD) is indicated by the dotted line

The majority of patients had a premorbid BMI SDS above the population average (i.e., BMI SDS = 0). The average premorbid BMI SDS for the whole group was 0,73 ± 1,00 (Fig. 1). The distribution of patients in the five premorbid BMI SDS classes is shown in Table 1. The patient in the higher premorbid BMI SDS classes had a higher BMI also at their prepubertal measurements. Moreover, from prepuberty to their top weight girls of the higher premorbid BMI SDS classes increased in BMI SDS while the opposite was true for the lower BMI SDS classes. The girls in the higher premorbid BMI SDS classes reached menarche at an earlier age than those in the lower BMI SDS classes.

**Table 1:** Anthropometric measurements, selected clinical and laboratory variables, and self-report instruments in 275 postmenarcheal adolescent girls presenting with an eating disorder

Premorbid BMI SDS class	1	2	3	4	5	*p*
N	11	60	100	79	25	
Prepubertal age (years)	7,5 ± 0,7	7,3 ± 1,0	7,3 ± 1,0	7,2 ± 0,8	7,4 ± 0,6	NS
Prepubertal BMI SDS	−0,73 ± 0,76	−0,12 ± 0,79	0,60 ± 0,85	1,31 ± 0,78	1,76 ± 0,82	<0,001
Age at menarche (years)	13,4 ± 1,12	12,7 ± 1,0	12,6 ± 1,0	12,3 ± 0,9	12,4 ± 0,8	*p* = 0,002
Age at documented top weight (year)	15,4 ± 1,4	14,7 ± 1,3	14,7 ± 1,3	14,3 ± 1,4	14,3 ± 1,7	NS
BMI SDS at documented top weight	−1,58 ± 0,62	−0,28 ± 0,47	0,62 ± 0,44	1,43 ± 0,35	2,35 ± 0,43	<0,001
BMI SDS change from prepuberty to top weight	−0,83 ± 0,64	−0,21 ± 0,78	0,01 ± 0,82	0,17 ± 0,76	0,68 ± 0,80	<0,001
Age at presentation (years)	15,8 ± 1,3	15,4 ± 1,4	15,6 ± 1,3	15,3 ± 1,4	15,7 ± 1,4	NS
Gynecological age (years)	2,2 ± 1,5	2,7 ± 1,4	3,0 ± 1,4	3,0 ± 1,3	3,3 ± 1,4	NS
Duration of eating disturbed symptoms (months)	9 ± 12	9 ± 9	10 ± 10	10 ± 9	12 ± 11	NS
BMI SDS at presentation	−2,79 ± 0,98	−1,60 ± 0,72	−0,61 ± 0,93	−0,13 ± 1,01	0,46 ± 1,11	<0,001
Weight loss (kg)	4,5 ± 3,8	5,0 ± 4,2	5,7 ± 4,9	9,5 ± 5,7	14,3 ± 7,9	<0,001
Rate of weight loss immediately preceding presentation (g/day)	74 ± 68	44 ± 62	31 ± 61	52 ± 66	60 ± 69	NS
Menstrual status (menses/amenorrhea/hormonal anticonception)	6/4/1	26/27/7	52/30/18	38/28/13	20/3/2	NS
	(55/36/9 %)	(43/45/12 %)	(52/30/18 %)	(48/36/16 %)	(80/12/8 %)	
Exercise as part of weight controlling behaviour	8 (73 %)	44 (73 %)	73 (73 %)	62 (79 %)	19 (76 %)	NS
Vomiting as part of weight controlling behaviour	1 (9 %)	16 (27 %)	33 (33 %)	20 (25 %)	7 (28 %)	NS
EDE-Q global score	1,7 ± 1,7	2,6 ± 1,5	3,3 ± 1,7	3,2 ± 1,6	3,1 ± 1,6	*p* = 0,012
EDE-Q eating concerns	1,7 ± 1,9	2,4 ± 1,8	2,9 ± 1,9	2,7 ± 1,8	2,6 ± 2,0	NS
EDE-Q restraint	1,3 ± 1,2	2,2 ± 1,4	2,7 ± 1,6	2,6 ± 1,5	2,6 ± 1,5	*p* = 0,021
EDE-Q shape concern	2,2 ± 2,2	3,2 ± 1,8	4,1 ± 1,8	4,0 ± 1,9	3,9 ± 1,8	*p* = 0,002
EDE-Q weight concern	1,8 ± 1,8	2,6 ± 1,7	3,4 ± 1,9	3,3 ± 1,8	3,2 ± 2,0	*p* = 0,011
MADRS-S	13 ± 11	16 ± 10	21 ± 11	17 ± 10	20 ± 12	*p* = 0,035
Diagnoses (AN/BN/EDNOS)	7/0/4	12/1/47	9/3/88	4/3/72	1/2/22	p < 0,001
	(64/0/36 %)	(20/2/78 %)	(9/3/88 %)	(5/4/91 %)	(4/8/88 %)	
Pulse rate	58 ± 9	61 ± 8	63 ± 7	62 ± 7	63 ± 8	NS
Systolic blood pressure	99 ± 8	105 ± 9	106 ± 10	108 ± 11	110 ± 10	*p* = 0,012
Plasma potassium (mmol/L)	4,3 ± 0,2	4,2 ± 0,3	4,2 ± 0,2	4,2 ± 0,3	4,3 ± 0,3	NS
Plasma phosphate (mmol/L)	1,18 ± 0,18	1,19 ± 0,17	1,19 ± 0,15	1,19 ± 0,15	1,21 ± 0,11	NS
Serum free triodothyronine (pmol/L)	3,5 ± 1,9	4,2 ± 1,3	4,2 ± 1,2	4,0 ± 1,2	4,0 ± 0,9	NS
Serum insulin-like growth factor 1 (μg/L)	227 ± 136	275 ± 116	308 ± 125	286 ± 132	310 ± 115	NS
Serum luteinizing hormone (IU/L)	0,43 ± 0,47	0,67 ± 0,74	0,89 ± 1,25	1,04 ± 1,53	0,86 ± 0,75	NS

Patients are grouped in one SDS wide classes according to the BMI SDS at their highest weight before the onset of the eating disorder. Class 1 represents premorbid BMI SDS

<−1,000, class 2 BMI SDS −1,000 - -0,001, class 3 BMI SDS 0–0,999, class 4 BMI SDS 1,000-1,999 and class 5 BMI SDS >1,999. Values are means ± SD. Statistical analyses are by one-sided ANOVA and Chi-square test for continuous and categorical variables, respectively

Reference values for laboratory parameters: Plasma potassium 3,7-4,8 mmol//L, plasma phosphate 0,94–1,63 mmol/L, serum free triiodothyronine 3,9–7,7 pmol/L, serum insulin-like growth factor 1 115–510 μg/L for age 12–15 years and 247–482 μg/L for age 16–20 years, serum luteinizing hormone >1 IU/L throughout the menstrual cycle but highly variable

*BMI SDS* BMI standard deviation score, *AN* anorexia nervosa, *BN* bulimia nervosa and subthreshold bulimia nervosa, *EDNOS* eating disorders not otherwise specified of restrictive subtype, *EDE-Q* eating disorders examination-questionnaire youth version; *MADRS-S* Montgomery-Åsberg depression rating scale-self report

At presentation of the ED age, gynaecological age and duration of ED symptoms did not differ between the premorbid BMI SDS classes. Those in the leanest premorbid BMI SDS classes were also the leanest at assessment. The patients in the higher premorbid BMI SDS classes were not underweight despite having lost more weight than those with lower premorbid BMI SDS. The rate of weight loss immediately preceding presentation did not differ between BMI SDS classes. Amenorrhea was reported in all premorbid BMI SDS classes and ranged from 37 to 51 % of the patients not on hormonal anticonception in premorbid BMI SDS classes 1–4. In the highest premorbid BMI SDS class only 13 % were amenorrhoic. Exercise as a means to regulate weight was common in all premorbid BMI SDS classes and about one third of the patients admitted to vomiting.

EDE-Q global score, the EDE-Q restraint, shape concern and weight concern subscale scores and the MADRS-S score differed between premorbid BMI SDS classes. For all these measures Tukey’s and Scheffe’s post-hoc tests showed that premorbid BMI SDS classes 2–5 formed homogenous subsets so that only BMI SDS class 1 differed from all the other classes.

A majority of 239 (85 %) patients had an EDNOS of a restrictive subtype. AN was more frequent in the lower premorbid SDS classes in which the girls presented with a lower BMI SDS.

Clinical and laboratory parameters at presentation did not differ between premorbid BMI SDS classes with the exception of systolic blood pressure which was lower in the lower premorbid BMI SDS classes. These parameters were further analysed by entering them as dependent variables against BMI SDS at presentation, weight loss and rate of weight loss at presentation in a stepwise linear regression analysis (Table 2). Pulse rate, systolic blood pressure and circulating hormones were all positively related to BMI SDS at presentation. There was also in varying degrees an inverse relationship with the magnitude of weight loss and the rate of ongoing weight loss at presentation. For those not on hormonal contraceptives (*n* = 234) secondary amenorrhea was entered as the dependent variable in a logistic regression analysis against BMI SDS, weight loss and rate at weight loss at presentation as independent variables. BMI SDS (odds ratio (OR) 2,49, confidence interval (CI) 1,77–3,51, *p* < 0,001 for a one unit difference) and weight loss (OR 3,09, CI 1,74–5,45, *p* < 0,001 for a 10 kg weight loss) independently predicted amenorrhea.

**Table 2:** Relationship between selected clinical and laboratory parameters and BMI SDS, total weight loss and rate of weight loss at assessment of 268 postmenarcheal adolescent girls presenting with an eating disorder

Dependent variable	Independent variables	Beta	R^2^	*p*
Pulse frequency	BMI SDS	0,326	0,188	<0,001
	Weight loss	−0,199	0,070	<0,001
	Final rate of weight loss	−0,189	0,027	*p* = 0,003
Systolic blood pressure	BMI SDS	0,311	0,152	<0,001
	Weight loss	-	-	-
	Final rate of weight loss	−0,249	0,056	<0,001
Plasma potassium	BMI SDS	-	-	-
	Weight loss	-	-	-
	Final rate of weight loss	-	-	-
Plasma phosphate	BMI SDS	-	-	-
	Weight loss	−0,141	0,020	*p* = 0,030
	Final rate of weight loss	-	-	-
Serum free triodothyronine	BMI SDS	0,191	0,033	<0,001
	Weight loss	−0,347	0,267	<0,001
	Final rate of weight loss	−0,315	0,107	<0,001
Serum insulin-like growth factor 1	BMI SDS	0,260	0,073	<0,001
	Weight loss	−0,245	0,048	<0,001
	Final rate of weight loss	−0,333	0,275	<0,001
Serum luteinizing hormone	BMI SDS	0,210	0,086	*p* = 0,003
	Weight loss	-	-	-
	Final rate of weight loss	−0,227	0,040	*p* = 0,001

The dependent variables were entered against BMI SDS, weight loss and the rate of weight loss immediately preceding assessment in a linear stepwise regression analysis

*BMI SDS* BMI standard deviation score

## Discussion

The data confirm the hypothesis that adolescents with restrictive ED and a high premorbid BMI are less lean at presentation despite a greater weight loss [10]. The important notion is that they nevertheless can have ED cognitions at the same level as more emaciated patients and have signs and laboratory findings of starvation despite near-normal weight. They may thus be as treatment requiring as emaciated patients with AN [17].

Signs of starvation such a low blood pressure and low heart rate are related to low weight [24]. As criteria for medical instability they are used for decisions on medical interventions and hospitalization [25]. The present data indicate that the severity of these signs depend on not only the low weight/BMI but also on the magnitude and speed of weight loss. This forms a parallel with observations of prolongation of the QTc interval, a risk indicator for cardiac arrhythmia, which is related to both low BMI and to the rate of weight loss [26]. The importance of the (rate of) weight change, and not only weight/BMI in absolute terms, for the medical status is further underscored by the laboratory findings, which confirm previous observations [27, 28] of the impact of ongoing weight loss on the endocrinological profile. This profile is heterogeneous [29] and some girls may be amenorrhoic even at an average weight [30]. Altogether, both the magnitude and rate of weight loss contributes to medical instability and cardiac risk [31, 32]. This emphasizes the need to carefully assess the weight history at presentation of an ED and not only register a point measure of weight.

The EDE-Q scores do not indicate that the girls in the higher premorbid BMI SDS classes, who presented with a higher BMI, had less eating disturbed cognitions compared to those with an average premorbid BMI. Compensatory behaviours were as prevalent as in the leaner premorbid weight classes and their greater weight loss suggests an even more restrictive eating. Just because they present with a near average weight they cannot be regarded as having a less severe ED. They may indeed be more entrenched in ED behaviours due to a greater and in some cases more rapid weight loss. This view is supported by the the observation in young adult women with AN that both low BMI and high weight suppression is related to ED psychopathology [14].

It is a novel observation that there is an increase in BMI SDS from before puberty up to a top measurement obtained post menarche in the higher premorbid BMI SDS classes. There is no difference in age/gynaecological age to explain this difference. It is likely that a high BMI confers some degree of body dissatisfaction. In combination, the high BMI and dissatisfaction with body shape and weight drives attempts to diet [33]. Even by the use of extreme weight control measures the desired weight loss is not achieved. Instead there is a further long-term weight gain, which could maintain dieting behaviours and confer an increased risk of developing an ED [2, 3]. This suggests that ED cognitions and extreme weight control behaviours may have been present in the highest premorbid BMI SDS class even before the time point reported as the onset of the ED.

Girls in the lowest premorbid BMI SDS class were very lean at presentation. This strengthens the concept of a relationship between premorbid BMI and BMI at presentation [10], especially so since the present data is based on documented measurements of weight and stature. These girls are constitutionally lean; their growth charts show that they have grown along low weight trajectories but have nevertheless achieved a pubertal growth spurt and reached menarche. This indicates that they are not always as medically compromised as their low BMI would suggest. In support of this is the observation that several are not amenorrhoic and that vital and laboratory parameters at presentation differ only little compared to the less emaciated girls in the higher premorbid BMI SDS classes. These very lean girls had unexpectedly low EDE-Q scores. It was, however, evident that some of them had a feeding/eating disorder with problematic eating behaviours but a less intensive drive for thinness or fear of weight gain. They would be diagnosed with EDNOS and score low on the EDE-Q despite a weight which would qualify for AN. Furthermore, scrutinizing the case records of the leanest premorbid BMI SDS class showed that there were several patients with a mismatch between the low EDE-Q scores and the description of ED cognitions/behaviours at the initial interviews. It therefore appears that in some cases denial or minimization of ED cognitions contribute to low EDE-Q scores in this group.

The strength of the present study is foremost the availability of objective measurements of premorbid weight in a sizable and unselected sample of adolescents with ED. Moreover, the availability of care and absence of doctor’s delay does not confound the analyses by adding to the duration of disease and magnitude of weight loss. There is a short-coming in the use of EDE-Q rather than the EDE interview. Although there is agreement between the two instruments [34] the EDE-Q may generate distortion due to minimization or denial as indicated above.

## Conclusion

In community samples of adolescents (subthreshold) bulimia nervosa is the ED usually associated with overweight [35]. The present investigation clearly demonstrates that restrictive ED may well be present among overweight adolescents. This may be overlooked when overweight adolescents are complimented on successful attempts to diet and reinforced by approving comments from family and peers. Indeed, the onset of weight loss may not necessarily be the onset of the ED. It is nevertheless important to consider the possibility of an ED when overweight adolescents lose weight rapidly. When assessing an ED in such adolescents a careful history of weight change and the development of ED cognitions is necessary in order not to underestimate the medical and psychological severity of disease.

### Ethics

All participants and their respective guardians consented to participate in the study. The protocol was approved by the Ethics committee of the Faculty of Medicien of Uppsala University.

### Availability of data and materials

Data will not be made available, it is to be included and further studies of follow-up of treatment.

### Consent to publish

Not applicable.
